# Comment: “Evaluation of the Association of Omentin 1 rs2274907 A>T and rs2274908 G>A Gene Polymorphisms with Coronary Artery Disease in Indian Population: A Case Control Study”

**DOI:** 10.3390/jpm10040190

**Published:** 2020-10-25

**Authors:** Karani S. Vimaleswaran

**Affiliations:** Hugh Sinclair Unit of Human Nutrition, University of Reading, Reading RG6 6DZ, UK; v.karani@reading.ac.uk; Tel.: +44-(0)-118-378-8702

**Keywords:** omentin gene, SNP, rs2274907, Hardy–Weinberg equilibrium, ARMS-PCR

## Abstract

The study by Jha et al. (2019) demonstrated an association of the single nucleotide polymorphism (SNP) rs2274907 A>T with coronary artery disease (CAD) in 100 CAD patients and 100 matched healthy controls from a South Indian population. There are serious concerns with regard to the interpretations of the study findings. The genotypes of the SNP are not in Hardy–Weinberg equilibrium (HWE) in both cases (*p* < 0.0001) and controls (*p* = 0.006), which is indicative of a technical error due to a problematic genotyping method. In addition, the genotype and allele frequencies reported in the study do not match with the frequencies listed in the SNP database for Asian Indians. While the study by Jha et al. reported ”T” allele as the minor allele, the dbSNP database reported ”A” as the minor allele. In summary, it can be concluded that the data presented in the study suffer from genotyping as well as data interpretation error and, hence, the findings should be considered by the reader with caution.

The Hardy–Weinberg equilibrium (HWE) test is commonly used for quality control of genotyping and is one of the few ways to identify systematic genotyping errors [1]. Jha et al. [2] did not perform the χ^2^ goodness-of-fit test to test if the genotypes of the single nucleotide polymorphism (SNP) are in HWE. Analysis of the HWE using the genotype data provided by the author in Table 3 of their article indicates significant departure from HWE for the SNP in both cases (*p* < 0.0001) and controls (*p* = 0.006). Reanalysis by other methods including likelihood-ratio test statistic [3] and exact test [4] also indicates the absence of HWE. Deviation from HWE in random samples is indicative either of technical error due to a problematic genotyping method or a presence of, in rare instances, natural selection in favour of a particular genotype group. The SNP rs2274907 in the omentin gene is a commonly studied marker and showed no significant deviation from HWE in other populations such as Iranian, Caucasian, Pakistani and Polish and multi-ethnic genotype data curated in the SNP database (dbSNP). Furthermore, the genotype and allele frequencies reported in the study by Jha et al. [2] do not match with the frequencies listed in the SNP database (dbSNP) for Asian Indians (Gujarati Indians in Houston, Texas). While the study [2] reported ”T” allele as the minor allele, the dbSNP database reported ”A” as the minor allele. 

Despite having a small sample size (n = 200) and only one participant with the rare homozygous genotype (TT), the authors have used different genetic models (codominant, dominant and recessive) to interpret the findings from the study. Besides coronary artery disease (CAD), associations with other cardiometabolic disease outcomes have also been tested; but none of these associations were adjusted for any of the potential confounding factors. In summary, it can be concluded that the data presented by Jha et al. [2] suffer from genotyping as well as data interpretation error. The authors used Amplification Refractory Mutation System-Polymerase Chain Reaction (ARMS-PCR) as their genotyping platform. As in the ARMS system, the design of compatible primers for simultaneous amplification is often challenging, and it has been shown to increase the probability of errors. A recent study [5] highlighted the inefficacy of the ARMS-PCR, where this technique failed to identify the amplification of the polymorphic allele. Some of the most common causes for the inefficacy of the technique include hairpin loop formation, annealing with the other primer (primer dimer) and dramatic difference in annealing temperature of each primer.

Any phenotypic–genotype association demonstrated by using such quality of genotype data is not reliable. So, the association of omentin SNP with CAD in Asian Indians presented by Jha et al. [2] should be considered by the reader with caution. An improved reliable genotyping method such as sequencing followed by genetic disease model-based HWE test should be used in the context of a case–control association study for complex disease outcomes.
